# “I don’t know if we’ll ever live in harmony”: a mixed-methods exploration of the unmet needs of Syrian adolescent girls in protracted displacement in Lebanon

**DOI:** 10.1186/s13690-021-00696-z

**Published:** 2021-10-09

**Authors:** Colleen M. Davison, Hayley Watt, Saja Michael, Susan A. Bartels

**Affiliations:** 1 Department of Public Health Sciences, Queen’s University, 62 Fifth Field Company Lane, Carruthers Hall #203, Kingston, Ontario Canada; 2 Department of Global Development Studies, Queen’s University, Kingston, ON Canada; 3 Bachelor of Health Sciences Program, Queen’s University, Kingston, ON Canada; 4 ABAAD Resource Centre for Gender Equality, Beirut, Lebanon; 5 Department of Emergency Medicine, Queen’s University, Kingston, ON Canada

**Keywords:** Refugee health, Adolescent health, Global health, Health care needs

## Abstract

**Background:**

The current crisis in Syria has led to unprecedented displacement, with neighbouring Lebanon now hosting more than 1.5 million conflict-affected migrants from Syria. In many situations of displacement, adolescent girls are a vulnerable sub-group. This study explores and describes the self-reported unmet needs of Syrian adolescent girls who migrated to Lebanon between 2011 and 2016.

**Methods:**

This mixed-methods study focusing on the unmet needs of adolescent girls was part of a larger research project on child marriage among Syrian migrants in Lebanon. Participants were recruited using purposive sampling in three field locations in Lebanon by locally trained research assistants. One hundred eighty-eight Syrian adolescent girls chose to tell qualitative stories about their own experiences. Using handheld tablets and an application called “Sensemaker” stories were audio-recorded and later transcribed. Participants were asked to then self-interpret their stories by answering specific quantitative survey-type questions. Demographic information was also collected. NVivo was used to undertake deductive coding of the qualitative data using Maslow’s Hierarchy of Needs as an analytic frame.

**Results:**

Among the 188 self-reported stories from adolescent girls, more than half mentioned some form of unmet need. These needs ranged across the five levels of Maslow’s Hierarchy from physiological, safety, belonging, esteem and self-actualization. Nearly two thirds of girls mentioned more than one unmet need and the girls’ expressed needs varied by marital status and time since migration. Unmet esteem needs were expressed in 22% of married, and 72% of unmarried girls. Belongingness needs were expressed by 13% of girls who migrated in the last 1–3 years and 31% of those who migrated in the previous 4–5 years.

**Conclusion:**

Many needs of displaced Syrian adolescent girls remain unmet in this situation of now protracted displacement. Girls most commonly expressed needs for love and belonging followed closely by needs for safety and basic resources. The level and type of unmet need differed by marital status and time since displacement. Unmet needs have been associated elsewhere with physical illness, life dissatisfaction, post-traumatic stress, depression, anxiety and even death. These results can inform integrated interventions and services specifically targeting adolescent girls and their families in the protracted migration situation now facing Lebanon.

## Background

The Syrian crisis began in March 2011 as a result of conflict between opposition groups and government forces [1]. The sustained conflict and declining safety in Syria have brought unprecedented displacement [1–3]. As of December 1, 2019, there were a total of 5,664,202 registered Syrian refugees, approximately 900,000 of whom have sought refuge in Lebanon [2, 3]. In 2015, a change in Lebanese immigration policy directed the United Nations High Commissioner for Refugees to cease registering refugees [4], and as such, there are also many unregistered migrants. Since 2011, the government of Lebanon has estimated that 1.5 million Syrians have been displaced into its country in total [5]. Many of these individuals face challenges in their everyday lives associated with their living conditions and the inaccessibility of basic services [6–8]. Although current data is limited, recent economic collapse, political instability, the 2020 massive harbour explosion and the COVID-19 pandemic in Lebanon are likely straining the lives of these migrant populations even further [ 9–13].

Based on data from the United Nations Department of Economic and Social Affairs, migrants aged 10–19 years made up 8.1% of the global migrant population and 16.6% of the displaced Syrian population in Lebanon from 2010 to 2019 [3]. Adolescents can be particularly vulnerable in situations of conflict and migration [14, 15]. Changes occur in their family and community roles, levels and types of responsibilities, and the presence and roles of adult advisors [16–18]. In situations of conflict and displacement, both male and female adolescent migrants often lack access to basic resources and services including food, clean water, safe and affordable housing, social support and healthcare [18–21]. In these contexts, adolescent girls commonly face additional specific challenges [22–27]. For instance, young female refugees have distinct needs for: basic health and social services; protections to ensure physical safety; and various forms of psychological support [21–23, 25, 26, 28]. Meeting these needs is crucial for them to thrive under the challenging circumstances of forced migration and in protracted refugee situations [ 18, 19, 21].

Maslow’s Hierarchy of Needs is a model developed by psychologist Abraham Maslow in the 1943 paper titled *A Theory of Human Motivation* [29, 30]. The model originally consisted of five stages of need or motivation associated with human development. These stages included: bodily physiology, safety, belongingness and love, esteem and self-actualization [31]. In subsequent writings [32, 33], Maslow added cognitive needs (primarily for those in academic pursuits), aesthetic needs (for artists) and a final stage of self-transcendence or personal spirituality. In general, Maslow subscribes that individuals fulfill lower level basic needs or motivations as foundations for achieving successive levels [31]. For instance, the main motivations for infants are physiological. As these are largely met, motivation for personal safety becomes prominent. This model has been critiqued [34, 35], but also widely used to guide understanding of human development and motivation in a variety of contexts [36, 37]. In this study, we apply Maslow’s Hierarchy of Needs to understand and describe the needs and motivations expressed by adolescent female Syrian migrants displaced to Lebanon between 2011 and 2016*.* The aim of this project was to listen to the stories of adolescents, consider and summarize the needs they expressed and echo their voices to inform interventions and services in the Lebanese humanitarian context.

## Methods

### Data collection

This project is part of a larger study of child marriage and the experience of Syrian girls in Lebanon. The survey was pilot tested in May 2016 and mixed methods data were collected on handheld tablets in three geographic locations across Lebanon between June–August 2016. An application called Sensemaker™ [ 38] was used to collect qualitative (audio-recorded stories) and follow-up quantitative questionnaire data. There were 1432 Syrian and non-Syrian individuals in the larger study. Twelve Syrian or Lebanese research assistants were trained for data collection, half of those trained were women. Purposive, convenient sampling was used. Recruitment was conducted in public locations and through the contact networks of the implementing partner- the ABAAD Resource Centre for Gender Equality. The sample for the larger study was amassed from participant groups that included: married and unmarried girls, mothers, fathers, husbands or unmarried men, and community leaders. After providing information about the study and obtaining informed consent, participants were asked to share any story they chose about the experiences of Syrian girls in Lebanon. Stories were audio recorded and following this, participants self-interpreted their story by answering a series of survey and demographic questions. Further details about the methodology and findings from the larger project are provided elsewhere [39, 40]. The current study examined the stories provided by 188 Syrian adolescent girls aged 13–17 years who told stories of their own experiences and had displaced to Lebanon within the 5 years previous to data collection.

### Data analysis

#### Sensemaker survey items

Demographic information (age, time since migration, marital status, parental status, religion and current location in Lebanon) for the 188 adolescent girls who provided first person stories were accessed from the quantitative Sensemaker survey data. Basic descriptive statistics (sub-group counts and frequencies) were calculated.

Responses for three questions from the survey were also analysed including: (1) “The shared story mostly relates to...?”; (2) “Based on the experiences shared, what is needed to improve life for Syrian girls in Lebanon” and (3) “Based on your story, what is the importance of [specific factors] for Syrian girls and their parents”. Questions 1 and 2 were asked in the form of a ternary plot or “triad” meaning that respondents were asked to place the indicator at a point in a triangle with three possible response options. Respondents could choose to place their response anywhere within the triangle, thus allowing them to respond towards an extreme single item, or anywhere between two items or centrally to indicate some combination of the three items. These data are summarized visually with the culmination of all 188 responses on a single triangle using the data visualization tool Tableau. Visual inspection of the summary triad helps to identify potential patterns in the responses across the group of participants.

For the triad asking about what could help improve the lives of Syrian girls, the data was disaggregated into two subgroups: married girls (current or previously married) and unmarried girls. To statistically determine if average responses might be different between the two groups, a three-part location coordinate was determined for each individual response relative to their location to the three vertices. A geometric mean and 95% confidence ellipse for each group were then calculated (using an R statistical macro). The geometric mean is used for data that are bounded by a constant-sum/closure constraint (since being closer to one of the vertices means you must in turn be farther from another). The 95% ellipse represents a statistical estimate of the boundary in which we expect the mean for a particular group or cohort to fall. If two 95% confidence ellipses do not overlap, then the means for each group are considered different. Specific details about these methods are provided elsewhere [41–44].

The third question was asked in the form of a rectangular plot or “canvas”. Participants could select various “stones” (possible response items) and place them on the canvas with x and y axes of “importance to the parents” and “importance to the girl” respectively. These axes run from zero/no importance (at the x/y intersection) outwards, indicating increasing importance as the axis extends out. This question was summarized in visual form using Tableau, with all responses for two potential response items (happiness and safety) being placed on a canvas. Visual inspection of this summarized canvas helps us determine potential patterns of response across the group of girl participants.

#### Qualitative Analysis

Audio recorded stories were transcribed and translated from Arabic to English. All transcripts were uploaded into NVivo qualitative analysis software. Coding was undertaken by two researchers (CD and HW). To begin, all 188 stories were read and independently deemed to include mention of any kind of unmet need, or not. These decisions were discussed and a group of stories (*n* = 84) were omitted from further qualitative analysis due to lack of information relevant to the main research question. Next, the remaining 104 stories were template coded by both coders using codes for the five levels of Maslow’s Hierarchy of Needs. Any discrepancy in coding was discussed and reconciled prior to proceeding. As a final analytic stage, the selected quotations for each of the five levels of needs were compiled into separate files. A third round of open-coding was applied to each of these files elucidating patterns and sub-categories of content within each level of need. Narrative summaries of the findings were then written.

## Results

An overview of the demographic characteristics of the study sample of Syrian refugee adolescent girls is provided in Table 1.

**Table 1:** Description of the Study Sample: 13–17 year old Syrian Adolescent Girls Displaced in Lebanon (*n* = 188)

	Counts	
Characteristic	*n*	*(col %)*
Location in Lebanon		
Beirut	39	(20.7)
Tripoli	56	(29.8)
Beqaa Valley	93	(49.5)
Years in Lebanon		
< 1 yr	16	(8.5)
1–3 yrs	61	(32.4)
4–5 yrs	111	(59.0)
Marital Status		
Single	112	(59.6)
Married	68	(36.2)
Widowed/Divorced/Separated	7	(3.7)
Prefer not to Say	1	(0.5)
Parental Status		
Yes - Parent	38	(20.2)
No - Not a Parent	146	(77.7)
Prefer not to Say	4	(2.1)
Religion		
Sunni	181	(96.3)
Druze	1	(0.5)
Alawite	1	(0.5)
Prefer not to Say	5	(2.7)

**Fig. 1:**
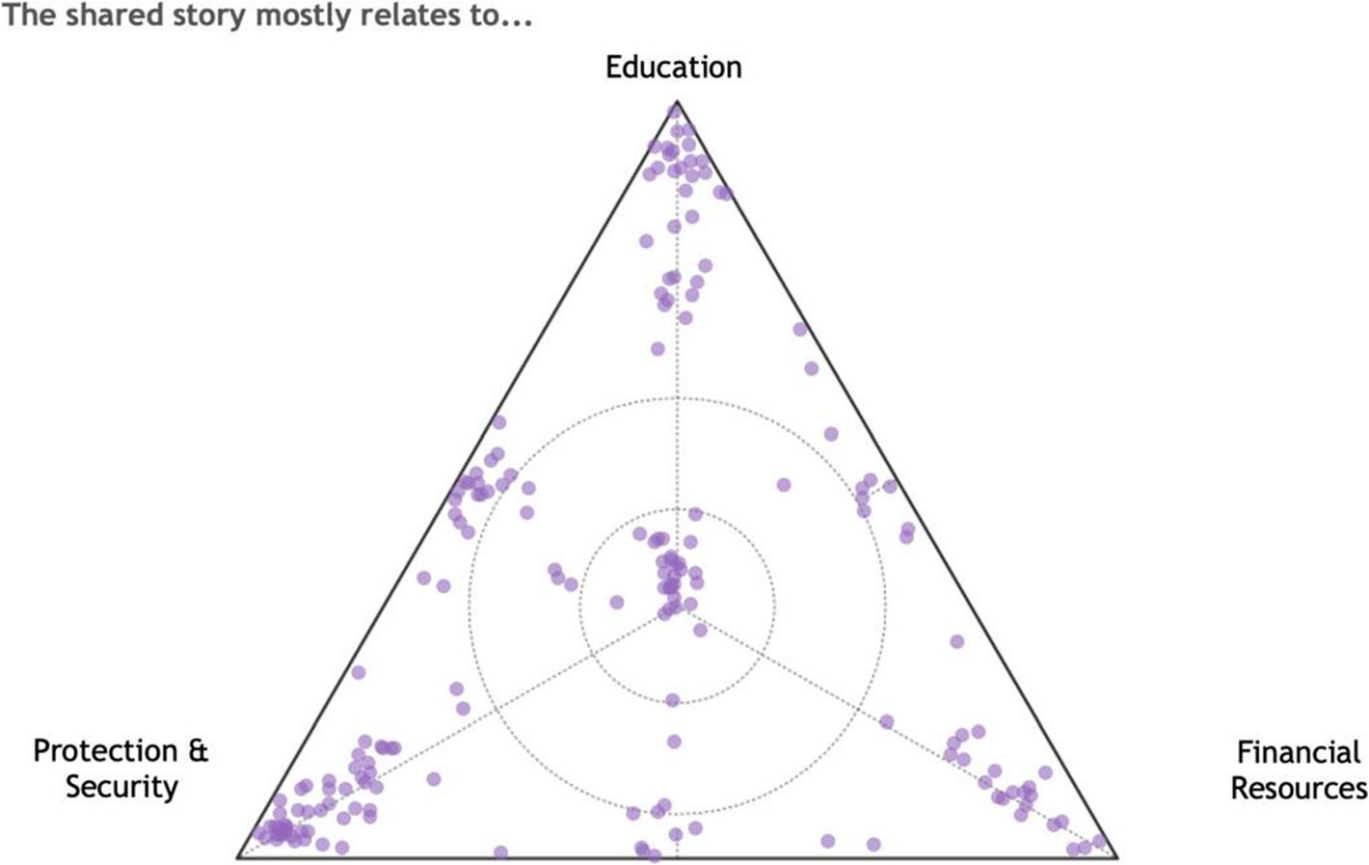
The Topic Area of First-Person Stories Shared by Syrian Adolescent Girls (aged 13–17), *n* = 188

When asked to indicate what their story was mainly about, the adolescent girls gave diverse responses noting that their stories touched on the themes of education, safety and financial security, with some girls indicating a combination of themes (Fig. 1). Visual inspection of the triad indicates greatest density of responses at the security item, with a noted grouping at education as well. There are obvious patterns indicating responses that pick up on a combination of items, especially the combination of education and security and the central location indicating a combination of security-education-financial security as well.

### A description of unmet needs

Among the 188 girls in the study aged 13–17, there were 104 (55%) who specifically mentioned some form of unmet need. Participants indicated needs associated with all of the levels in Maslow’s hierarchy, but in varying degrees and with important sub-categories.

#### Basic needs

Basic needs are defined as the prepotent biological and physiological needs of human organisms, including: food, hydration, shelter, warmth, sanitation and sleep [30]. Thirty-eight of the 188 stories (20%) spoke specifically about an unmet need at this level, or about one in four (27%) of the 104 adolescents who discussed any unmet needs.

Unmet needs for housing and sanitation that were identified by the Syrian girls were primarily related to the conditions in refugee camps and informal tented settlements. Many girls described crowded dwellings, with many family members or multiple families living in a single tent. This is depicted in the statement:

*“We used to live with our father in a house, now we live with a nation in a tent.”* [ID507]

The conditions are further described as inappropriate and uncomfortable, with limited access to basic resources such as clean water for hydration and sanitation for themselves and their children, for example:

*“...The tent is inappropriate, it’s too hot, and I live with my two little kids in one room, and it’s full with mice and snakes and insects … We are paying the rent of a small tent, and there is no water, neither water for drinking nor water for cleaning, we go too far places to get water, and it is too hot, my kids are getting too many times sick because of this problem.”* [ID253]

*“Here we pay for everything, we pay for water, we might even have to pay for the air that we breath someday, my husband can't get us enough money, he's overwhelmed by the amount of money that he has to raise. I once even got sick and suffered a lot, being a foreigner is hard.”* [ID868]

In some cases, girls viewed marriage as a means to achieve financial stability to meet at least basic needs:

*“We don’t have money for food. I want to get married to have a better life. We need money. I need to get married to be able to get what I need.”* [ID820]

#### Safety needs

Safety needs relate to personal security, stability, protection, law, and freedom from fear [30, 31]. There were 46 adolescent girls who mentioned unmet safety needs in their narratives. This was 25% of all the 188 girls in our study, and 44% of the 104 adolescent girls who spoke about any unmet needs at all. These deprivations were unmet needs for access to healthcare in cases of medical need, fear and insecurity associated with domestic violence, need for safe transportation, limited child protection and lack of access to formal paperwork or registration.

Unmet needs for healthcare were consistently discussed by the girls and from their perspective represent threats to individual safety. Girls stated that in some cases, they would attempt to travel back to Syria to access healthcare, even at risk to their own safety. These sample quotations highlight the safety issues associated with unmet needs for healthcare specifically:

*“I went to a hospital here, but no one helped us. I spent three days in the hospital in Saida [Lebanon], and no one helped us. The medical expenses were very high, and you are aware of our situation here. I went back to Syria to be treated.”* [ID1501]

*“... We do not have any medical care. Since going to a hospital costs a lot, if anyone was sick we don’t seek any medical help. The situation is not safe.”* [ID1238]

Domestic violence is an additional safety concern and was specifically identified by 10 of the 188 participants. These stories commonly associated violence with being married at a young age, for example:

*“I was 15 years old when I got married. I didn’t get engaged, and I didn’t do a wedding. We were married in four days only. Problems started immediately. I lived with him for 20 days only. He used to beat me, ...”* [ID271]

Safety needs also relate to the safety of the participants’ children. In some cases, the young mothers would sacrifice their own safety for the wellbeing of their children.

*“I had a daughter and he still beats me … I endured our abusive relationship for two years. I was patient for my daughter’s sake.”* [ID432]

Both married and unmarried Syrian girls mentioned feeling unsafe in their communities due to fear of violence towards them:

*“And here in the camp there is no safety, after it’s dark we can’t go out, and I’m under the 18 years, and if whatever happened to my kids, even if my kid dies, I can’t go out before my husband is back I don’t feel safe going out alone, after it is dark there is no safety.”* [ID253]

The safety needs identified are further exacerbated by a lack of formal paperwork and registration, which can impede movement and access to services, work and schooling. Again, girls spoke about putting themselves at risk by trying to travel back to Syria to renew papers:

*“Our papers need renewal and a guarantor. We tried to go to Syria in order to renew our papers there, but we were unable to go, since our permits are expired and the borders are closed.”* [ID665]

**Fig. 2:**
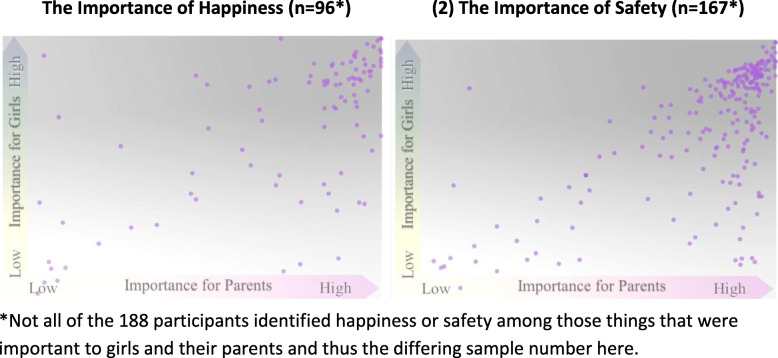
Girls’ Perceptions of the Importance of (1) Safety and (2) Happiness for them and for their parents. *Not all of the 188 participants identified happiness or safety among those things that were important to girls and their parents and thus the differing sample number here

When girls were asked what is important for Syrian girls in Lebanon, from their own and their parents’ perspectives, girls list safety and happiness among those things most important (Fig. 2).

#### Needs for love and belonging

Belonging and love needs are characterized by motivations for social wellbeing, belongingness, affection and love from family, friends, community groups or within romantic relationships [30, 31]. There were 51 girls who expressed specific unmet needs for belongingness and love, representing 27% of the overall sample and approximately half (49%) of girls who expressed any unmet needs at all. Many girls spoke about disturbed relationships and a loss of a sense of love and belongingness as a result of displacement. These needs were related to separation from family and friends by distance or borders, as well as feeling unsafe in public in Lebanon. Many of the girls discussed experiences of social isolation as they were often unable to safely go out, socialize or build new friendships. For example, one girl explains the multiple sources of social isolation and how this made her feel:

*“...My sister got married and I was left alone. It felt very lonely. Not one day would pass that I wouldn’t cry and hate my life. Shortly afterward, my mother left too and I was completely alone and it felt horrible.”* [ID1417]

Another participant details how the migration from Syria changed her loving relationships and the way her situation over time made her choose to get engaged even though she was uncertain about it:

*“I'm a woman who used to love my life in Syria, the love, the care, anyone would listen, anyone would help. We came here to Lebanon and we lost hope, trust and love. Each person wanted to solve their own problems, no one had the patience for other people's problems, I got engaged but not because I love him but just because I'm at an age where I should find the partner of my life. I can't seem to be happy or comfortable with him, there’s no harmony. but I only imagine the consequences if I leave him, society will judge me, it won't be accepted, I don't know if we'll ever live in harmony. I can't leave him now.”* [ID938]

Marriage appears to have improved some girls’ love and belonging although this is balanced by experiences of intimate partner and domestic violence and extreme unhappiness in marriage situations. See, for example, this set of disparate marriage relationship experiences that were shared by participants:

*“I decided to try to work and study at the same time...The curriculum started, but I only attended for 3 weeks. The principal interfered in what we had to wear, and he decided that girls and boys shouldn’t socialize together. I didn’t attend my classes anymore and continued to work. I liked my work and the people I worked with. The best thing that happened to me was that I met my husband here. His family is my family now.”* [ID1545]

*“I was forced into getting married, and I couldn’t get along with my husband. I was a teenager, and I was so young. I didn’t understand what marriage is. I couldn’t get along with them, neither with my husband nor with my parents in law.”* [ID432]

The quality of social relationships is hindered by negative aspects of the girls’ living environments, emotional stress and, in some cases, physical abuse and sexual violence. This is in a context of very limited or lack of peer and family psychological or social supports in Lebanon compared to their lives in Syria:

*“Sitting and being trapped inside the house is not a good situation. You feel the atmosphere is sickening. It is such a big difference from our life in Syria. We were living in big homes, a cleaner atmosphere, cleaner water and lots of differences. Even the social relations or social environment in Syria was a lot better; you were with your family, your uncle’s family, your friends – anywhere you go you would find people around you. But here, if you become sick, no one will come to your door. No one will even feel for you or notice.”* [ID355]*.*

Lack of feelings of love or belonging are also exacerbated for Syrian girls in Lebanon due to prevailing societal discrimination that hinder their integration:

*“I would cry a lot, especially from the insults. “You, Syrians, are coming to rob us” or “You, Syrians, are coming to torture us”. These curses and insults bother me a lot, and I’d feel choked, then I’d cry. I was going through a huge psychological pressure, and I wasn’t feeling comfortable.”* [ID1501]

#### Esteem needs

Esteem needs, as described by Abraham Maslow, involve human motivation for a stable and generally positive evaluation of ourselves, a sense of independence, status, and personal achievement [30]. This level of the hierarchy was the least represented among the unmet needs expressed by the girl participants. There were only 13 examples of specific mention of unmet esteem needs, representing 7% of the 188 total participants and 13% among the 104 who noted any unmet needs at all. Expressed esteem needs were principally related to the effects of discrimination the girls feel as foreigners in Lebanon and the influence that lack of access to education and income generating opportunities has on their sense of self. One participant explicitly states that harassment or “bullying” affects her self-esteem and the esteem of other Syrian migrants. Others describe the feeling they get because of being “Syrian” in Lebanon:

*“I hope that Lebanon treats Syrian better because we are all brothers and sisters. I also hope that the bullying stops as it affects our self-esteem.”* [ID348]

*“Of course our life was better there...if you're Syrian, they are disgusted by the word, when you say "Syrian" it's just as if you mentioned something "filthy", they treat you like an animal here, they don't give you any sort of value.”* [ID614]

Participants expressed needs for opportunities for education and opportunities for income generation, recognizing their links with personal financial stability, independence and feeling respected. Education remains largely inaccessible for many migrant Syrian girls in Lebanon due to the high costs of schooling, safety concerns, and lack of formal accreditation for Syrian students in Lebanese schools [45]. Many girls have wanted to continue their education but most have been unable to and this is affecting their sense of self:

*“I want to tell the story of how the situation in Lebanon and Syria is different, and how it has affected us for the worse, especially in our education. We were very hopeful of becoming recognized members in the community, but now, we do not have that hope anymore.”* [ID141]

#### Self-actualization needs

Individuals ideally have opportunities for personal growth, fulfillment and finding personal meaning in life. Thirty participants spoke about specific unmet self-actualization needs, representing 16% of the overall sample and about a third (29%) of the participants who expressed any form of need. The concerns expressed were largely related to achieving personal goals in education, finding employment to support their livelihood, and long-term personal safety. Discussion of career aspirations were often accompanied by recognition that these ambitions may not be possible:

*“My dream is to finish my education and become a doctor one day, but my dream's been killed.”* [ID663]

*“We are not comfortable at all, our futures have been destroyed and they remain a mystery. I hope that an opportunity will come where I can properly study because I love education and I want to become someone important in society.”* [ID558]

*“I think girls should continue their education. After she finishes her education, then she can think about marriage. She would be working and have completed her education and would have a better financial status.”* [ID1498]

The narratives also reflect the girls’ feelings that their lives have lost significant meaning and an ongoing desire for improvement to the situation and a better future:

*“My life [in Syria] had a lot of meaning, I had a lot of beautiful things. But not anymore.”* [ID1500]

*“I hope every girl gets to live her life, a life of her choice, following the right path. I hope that we could someday go back to Syria, so that we could live our lives, that every Syrian girl could live her life without suffering, I have suffered a lot.”* [ID688]

#### Multiple needs and the intersections with marriage and time since migration

Adolescent girls commonly spoke about more than one type of unmet need. Indeed, in 63% of cases, girls expressed multiple needs, which existed across the more basic stages (physiological and safety) as well as the higher stages of the hierarchy (belonging, esteem and self-actualization). In particular, unmet safety needs were frequently expressed in conjunction with other unmet needs with 45% of the girls speaking about safety and physiological needs, and 40% speaking about safety and needs for belongingness/love.

Unmet needs for belongingness and love varied based on the number of years spent in Lebanon as well as by marital status. Among the girl participants who migrated less than three years before the date of the survey, 13% expressed love and belongingness needs. This was 31% among those participants who had displaced in the 4–5 years before the survey. The frequency of these kinds of needs also varied between married and unmarried adolescent girls, with 67% of married girls and 28% of unmarried girls reporting needs for love and belonging. Some of the married girls indicated that marriage helped them meet needs for love and belonging, while others said that they had hoped marriage would help but that it did not. Some participants indicated that marriage and motherhood gave them some freedom, purpose and happiness, while others said that they were exposed to abuse and were unhappy in their marital situations.

**Fig. 3:**
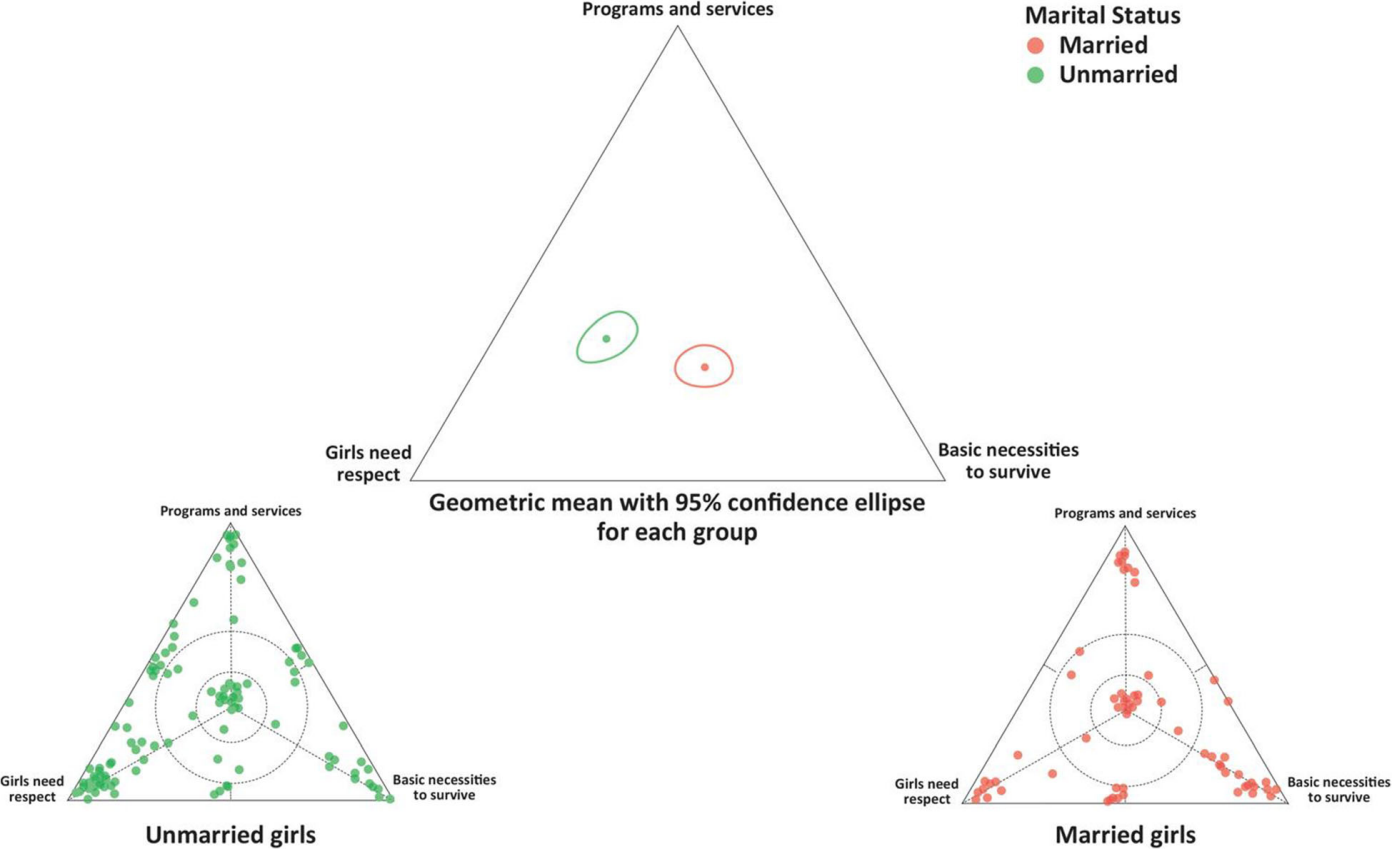
Girls’ opinions on what is needed to improve life for Syrian girls in Lebanon (unmarried *n* = 112 and married/widowed/divorced or separated *n* = 78)

When the group of participants was separated by marital status and the quantitative/ternary plot data examined, there were some similarities but also differences in response pattern. For example, when the girls were asked what was needed to improve the life of Syrian girls in Lebanon, both groups had average responses that were quite central but low in the plot. This indicates consideration of all three response items “basic necessities to survive”, “programs and services” and “girls need more respect”, but overall slightly less tendency towards answering that girls need programs and services. Married girls did tend to respond on average more towards “basic necessities to survive” than the unmarried girls and these differences were statistically significant (Fig. 3).

### Recognition of local efforts to address unmet needs

The participants in this study recognized formal and informal supports to address unmet needs. They spoke about organizations working to address needs at all levels of Maslow’s Hierarchy. For instance, while gaps do exist, NGOs are providing some access to shelter, food, clean water and basic health services. For some, there are safe spaces to socialize, opportunities for education and occupational outlets. The participants provide specific examples:

*“ABAAD (Resource Centre for Gender Equality) provided us with these activities which allowed us to express our potential to some extent.”* [ID1500]

*“We had to move to Lebanon because of the war, and I began work in a hair salon. At the moment, I am working with an NGO that takes care of camps, and if the association’s plan works, they promised to equip me with whatever I need to open my own hair salon.”* [ID534]

*“The UN is helping with food but rent is impossible and my daughter and husband need medical care.”* [ID1213]

## Discussion

In this study, migrant adolescent girls who were displaced from Syria to Lebanon between 2011 and 2016 were asked to tell stories about their life and experiences in Lebanon. Over half of the participants specifically mentioned unmet needs, with two thirds mentioning multiple unmet needs. The qualitative analysis of their stories indicates that at least some needs are not being met across *all* levels of Maslow’s Hierarchy, from basic and safety needs to needs for love, belonging, self-esteem and self-actualization. Our study contributes insight, from the girls’ perspective, on types of contextual needs across Maslow’s levels, simultaneous unmet needs and the fact that needs may differ based on time since displacement and with marital status.

Previous studies of unmet needs among adolescent girls and young women in situations of conflict and migration are relatively limited and have largely focused on specific needs associated with family planning, sexuality education and access to sexual and reproductive health services [ 46–48]. However, the connection between conflict-associated forced migration and mental health among adolescent migrants has been studied extensively showing similar and strong association between displacement experiences and poorer mental health. This is across diverse contexts including Darfur [49], Bosnia [50], Somalia [51], East Congo [52], previous studies in Lebanon [50] and others. In 2016, Ceri and colleagues [53] examined psychiatric disorders among Syrian and Iraqi Yazidi Kurd adolescents immediately following forced migration to Turkey. Very soon after displacement, adolescents experienced symptoms of disturbed sleep, acute and post-traumatic stress disorder, depressive disorder, and adjustment disorder among others [54]. The authors note that the participants in their study were all at a specific stage of migration (new arrival) and that symptoms, especially for stress disorders, would likely emerge and change as the new arrival stage transitioned into more protracted displacement and potential integration into the host country. Similar to these findings, the girl participants in our study showed some change in motivation and unmet need as time passed in Lebanon. Girls who had migrated 4 or 5 years previous to the survey expressed more needs related to love and belonging, for example, than girls who displaced more recently. Almost all of the girls spoke about being totally isolated, living in an environment where they do not feel safe to go out for fear of harassment or sexual violence and where they have no opportunity to make or keep friends. The participants spoke about a growing awareness of feeling unwelcome in Lebanon, experiences of discrimination and missing social opportunities and supports that they had enjoyed previously in Syria. The United Nations High Commissioner for Refugee defines protracted displacement as situations where refugees from the same nationality have been in exile for five consecutive years or more in a given host country [55]. We believe that as their displacement became protracted, the trauma and adversity the girls faced was not only associated with their original forced migration or early experiences in Lebanon, but with the experience and ideas of ongoing poverty, discrimination, and loss of hope for the future.

In Maslow’s theory of human motivation [29] he notes that basic needs and motivations must be met first before people focus on needs or motivations higher up in the hierarchy. Many service providers have also used the same rationale with arguments for providing “housing first” before mental health services [56], or food, such as breakfast programs, before educational pursuits [56]. The girl participants in this study often mentioned basic needs and how priority had to be placed on them. They spoke about giving up educational dreams to pragmatically address daily needs for food, shelter or medicine, for example. They also provided accounts of potentially risky trips returning to Syria when faced with health care needs in the case of emergencies or dire illness.

While basic and safety needs do have inherent importance, critiques of Maslow’s theory often focus on the fact that basic and security needs might ideally be met first, but in some situations, such as those of long-term poverty or temporary changes to provision of basic needs, humans do exhibit motivations beyond the basic levels. Tay and Diener [57] attempted to build construct validity around Maslow’s Hierarchy of Needs. They included data from 60,865 participants from 123 countries collected between 2005 to 2010. The results support the view that universal human needs do exist regardless of cultural differences. However, the conceptualization of basic and safety needs, at the base of the pyramid, being essential prior to higher order motivations was not supported. Instead, the authors noted that human needs were like vitamins, some might be more essential than others at any given time but basically, humans require all of them to fully thrive [57]. Similarly, the girls in our study expressed unmet needs at all levels of the hierarchy in a non-hierarchical fashion, as well as multiple needs expressed simultaneously. Even in cases where basic or security needs were unmet, girls expressed motivations for love, belonging, and self-actualization, for example. In most cases, the girls in this study had more secure and comfortable lives in Syria before displacement. In their previous contexts, it is likely that their basic and safety needs were not grave concerns, and girls would have almost universally attended school. In their situation of forced displacement, their desires for love and belonging, or self-actualization were not extinguished even though they now face unmet basic and safety needs.

The most common form of need expressed by the Syrian adolescent girls in this study were those of love and belonging. Nearly half of all the needs expressed were from this level of need. Girls spoke about their social isolation, lack of friends, lack of community support, missing family and their inability to safely go out into the community. A third of the participants who expressed love and belonging needs also spoke about needs related to self-actualization. When the girls’ situation changed in the process of displacement, the girls felt the loss of not only family, friends, school peers and social events, but also a future.

One potential explanation of these patterns lies in an understanding of adolescence as a developmental stage, and the importance of social relationships particularly at this time of life. Age differences in expression of needs and motivations have been studied previously. Nearly forty years ago, Goebel and Brown [58] looked at needs expressed by 111 people between ages 9–80 (53 females, 58 males) including 21 school-going Caucasian adolescents in the United States with an average age of 15 years. Children 11 years and under most commonly expressed basic needs. Love and belonging needs were most prominent among the adolescents with esteem needs also peaking in adolescence. Our data with Syrian girl migrants indicate that love and belonging was commonly mentioned, but self-esteem was not. Esteem needs were the least commonly expressed motivations across any situations perhaps highlighting a de-emphasizing of self among our participants. Goebel and Brown [58] did note sex differences, stating that the young women in their study had lower overall frequency of esteem needs as compared to boys of the same age. Pope and colleagues [59] noted that low self-esteem results when there is a large discrepancy between self-image and ideal self. Because we did hear numerous times that girls were sad about lost hopes for the future, or sudden changes in their educational opportunities, it is likely that self-image at the time of our study did not meet ideal self for many of the girls. In addition, some early theorists [60] place the origin of self-esteem in the social realm. The girls in our study often spoke about social isolation and sadness associated with a loss of friends and relationships. In a previous study of migrant girls in Turkey [61], the adolescents encountered a variety of challenges including peer relations, discrimination, bullying, adaptation and poverty that impacted their self-esteem in complex ways. The girls in our study described a context of discrimination, insecurity and isolation and also provided many accounts of gender-based violence and inability to go out. It is possible that these girls did not immediately turn to stories of self-esteem specifically, but instead chose to express their concerns about the future or about social relationships. While not specific to their sense of self, even telling these stories, in a context where speaking about these hardships may be somewhat taboo, shows strength of self.

The high frequency of mention of love and belonging needs among the girls in this study is a key issue that should be addressed by programmatic and policy efforts. According to the participants in our study, the day to day lives of Syrian girls in Lebanon are lonely and full of fear and stress. Previous research indicates that social connections can be some of the strongest supports to mitigate pre- and post-migration stress [62, 63]. A sense of social support and belonging has also been positively associated with self-esteem, self-efficacy and life satisfaction [64, 65] as well as being protective against poor mental health and the effects of stress [66]. Refugees have themselves identified a sense of belonging as key for successful integration in a host community [67–69]. Thorne [70] worked with six former adolescent refugees and explored their ideas about belonging. Qualitative interpretive description was used and the author identified five pathways to belonging from the migrants’ perspective. These were: (1) Feeling comfortable, (2) Feeling confident, (3) Feeling accepted, (4) Having a sense of purpose, and (5) Integration. The lived experiences of discrimination and social isolation in Lebanon that were described by the Syrian girls in our study makes it easy to understand why these migrants do not feel a sense of belonging, and why their love and belonging needs remain unmet. Previous research focusing on migrant adolescents and their struggles to belong highlights hostility and exclusion across diverse national contexts. Young migrants’ sense of belonging is often mediated by processes of racialization and “othering” in the host country [71]. Jetten, Haslam, and Haslam [72] note that building a sense of social inclusion and belonging is a critical “blind spot” among practitioners, theorists, and the general public. Further support for safe spaces for adolescent girls to congregate and socialize, improved community-level security so girls can go out into the community without fear of violence and safe and accessible educational opportunities for girls are key points of continued intervention in the now protracted displacement situation for Syrian migrants in Lebanon.

Our findings highlight the importance of basic needs, yet, concurrently stress the significance of other higher-level needs for girls especially in prolonged displacement. This is in line with Maslow’s indication that hierarchies are interrelated rather than sharply separated [73], which is vital in informing and shaping humanitarian response efforts. For instance, despite the importance of basic needs at the onset of a crisis, attending to this need alone by humanitarian aid is not sufficient to ensure refugee dignity and overall well-being. Findings from a study conducted in a Jordanian camp populated by Syrian refugees similarly presents the expressed need for programming that additionally addresses higher-level needs such as belonging and self-esteem linked to a sense of contribution or value [36]. An effective and efficient humanitarian response that places relationships and human dignity at its core should therefore attend to the interlinkages of Maslow’s needs instead of approaching them in a singular or even stepwise fashion.

### Implications for public health intervention

The findings of this study indicate that displaced Syrian girls have specific needs that are not being met and could be further supported by specific targeted public health interventions as they navigate the current reality of protracted displacement in Lebanon. While both governmental and non-governmental agencies have been providing food, water, shelter, medical services and safe spaces for some displaced Syrians since 2011, many basic needs still remain. In addition, Syrian migrant girls expressed ongoing needs at higher motivation levels such as those related to love, belonging, esteem and self-actualization, which should be simultaneously addressed.

The required public health interventions are varied and many. Basic needs are essential for human survival and the fulfillment of human [74] and child rights [75] and must remain priorities. Lebanon is a signatory to the Universal Declaration of Human Rights which includes article 25 outlining the right to a standard of living adequate for health and well-being including food, clothing, housing, medical care, necessary social services, and the right to security [74]. The UN Convention on the Rights of the Child was also ratified by Lebanon in 1990 and includes a commitment to the provision of food, clothing, a safe home (article 27) and education (article 28) among other rights for children [75]. There is also specific confirmation of the rights of refugee children (article 22) and the protection of children in situations of war (article 38). Unfortunately, Lebanon has not signed the rights conventions pertaining to refugees or stateless individuals [76]. The state is currently experiencing a profound socio-political and economic crisis with growing poverty, failure of even basic services and dire depletion and mismanagement of human, natural and financial resources [77]. Previous scholars and critics note that from a government perspective, displaced Syrians are largely seen as temporary visitors to whom there is minimal, if any, state obligation [78–80]. In this context, the UN and other non-governmental agencies play a key role in the provision of services for displaced Syrians. Supports continue to remain critical as the length of the protracted displacement grows and needs of displaced populations remain and shift. Our own study indicated that the needs of Syrian girls in Lebanon were different depending on how long the girls had lived in the country. For example, needs for love and belonging grew as time in Lebanon lengthened. This highlights the growing need for humanitarian response actors to routinely monitor such shifts in needs, while practicing flexibility in programmatic design to ensure these diverse needs are being met.

In addition to interventions that ensure the continued provision of basic needs, it is critical that the humanitarian response adopt a holistic multi-sectoral vision that aims to provide safe spaces for Syrian girls to socialize, learn and be able to envision and create viable and healthy futures for themselves. For instance, targeted, gender specific programs that provide educational and occupational opportunities are essential, alongside initiatives that promote peer and social support. Moreover, population level campaigns to improve the acceptance and integration of displaced Syrians in the daily lives of host communities in Lebanon are also required, especially noting the recently worsening social and economic situation in the country. Humanitarian aid and emergency response in Lebanon is facilitated through such mechanisms as the Humanitarian Program Cycle (HPC) [81] and the Cluster System [82]. Our findings support inter-cluster response and operational coordination, encouraging different cluster actors, such as nutrition, protection, shelter and education, to work together to support migrant populations, including girls. Lastly, because many displaced Syrians in Lebanon do not have formal refugee status, it is important that any services remain inclusive regardless of legal status.

### Study strengths and limitations

This study includes the stories of migrant adolescent girls, whose voices are often missing or silenced, speaking about their own experiences. The girl participants were residing in three areas of Lebanon where the living conditions and experiences of migrants can differ and we believe having this diversity in the sample lends strength to the study. Sensemaker is a data collection tool that allows for the collection of narrative stories as well as participant self-interpretation and socio-demographic survey data. This combination of qualitative and quantitative data provides both depth and breadth in the data collection. While we did have access to the girls own stories, we were not able to also assess these responses by specific age (as all participants were recorded as being between ages 13–17). It is possible that the married girls were slightly older by comparison. In addition, because the sample was not randomly selected, there is likely some response and participation bias. Girls who were able or interested in responding to the survey, may have differed systematically from those who were not able or not interested in participating. If girls who were unable to participate experienced even greater social isolation and unmet basic needs, the results we found may be an under representation of the problems.

## Conclusion

The level of continued unmet need associated with basic resources such as food, water, adequate shelter and personal safety is greatly concerning. These data were collected prior to the significant economic collapse and political instability in Lebanon since 2016, as well as the COVID-19 pandemic. It is very likely that the level of basic need among Syrian migrants has increased substantially in recent times [9–13]. Despite their critical nature, basic needs must be seen to intersect with other levels of Maslow’s hierarchy including safety, belonging and esteem. The girls themselves told us that they do not feel love and belonging in Lebanon and that they desire some positive options for the future. Any long-term solution for them must take into account both their basic needs as well as their higher needs and motivations, allowing them to find ways to belong and reach their own potential. Comprehensively attending to the diverse needs identified is essential to ensuring the dignity of displaced communities in general and girls more specifically.

## Data Availability

Requests for access to the de-identified dataset for this study can be made through the corresponding author.

## Abbreviations

HPC: Humanitarian Program Cycle
NGO: Non-governmental organization
UN: United Nations
